# Mammographic density change in a cohort of premenopausal women receiving tamoxifen for breast cancer prevention over 5 years

**DOI:** 10.1186/s13058-020-01340-4

**Published:** 2020-09-29

**Authors:** Adam R. Brentnall, Ruth Warren, Elaine F. Harkness, Susan M. Astley, Julia Wiseman, Jill Fox, Lynne Fox, Mikael Eriksson, Per Hall, Jack Cuzick, D. Gareth Evans, Anthony Howell

**Affiliations:** 1 Centre for Cancer Prevention, Wolfson Institute of Preventive Medicine, Queen Mary University of London, Charterhouse Square, London, EC1M 6BQ UK; 2 Department of Radiology, Addenbrooke’s Hospital, University of Cambridge, Cambridge, UK; 3 Division of Informatics, Imaging and Data Sciences, School of Health Sciences, Faculty of Biology, Medicine and Health, University of Manchester, Manchester, UK; 4 Prevent Breast Cancer Centre, Wythenshawe Hospital, Manchester University NHS Foundation Trust, Manchester, M23 9LT UK; 5 Manchester Breast Centre, Manchester Cancer Research Centre, University of Manchester, Manchester, M20 4BX UK; 6 Department of Medical Epidemiology and Biostatistics, Karolinska Institutet, Stockholm, Sweden; 7 NW Genomic Laboratory Hub, Manchester Centre for Genomic Medicine, Manchester University NHS Foundation Trust, Manchester, M13 9WL UK; 8 Division of Evolution and Genomic Sciences, School of Biological Sciences, Faculty of Biology, Medicine and Health, Manchester Academic Health Science Centre, University of Manchester, Manchester, UK; 9 Division of Cancer Sciences, Faculty of Biology, Medicine and Health, University of Manchester, Manchester, M13 9PL UK

**Keywords:** Tamoxifen, Prevention, Mammographic density, Breast density change

## Abstract

**Background:**

A decrease in breast density due to tamoxifen preventive therapy might indicate greater benefit from the drug. It is not known whether mammographic density continues to decline after 1 year of therapy, or whether measures of breast density change are sufficiently stable for personalised recommendations.

**Methods:**

Mammographic density was measured annually over up to 5 years in premenopausal women with no previous diagnosis of breast cancer but at increased risk of breast cancer attending a family-history clinic in Manchester, UK (baseline 2010-2013). Tamoxifen (20 mg/day) for prevention was prescribed for up to 5 years in one group; the other group did not receive tamoxifen and were matched by age. Fully automatic methods were used on mammograms over the 5-year follow-up: three area-based measures (NN-VAS, Stratus, Densitas) and one volumetric (Volpara). Additionally, percentage breast density at baseline and first follow-up mammograms was measured visually. The size of density declines at the first follow-up mammogram and thereafter was estimated using a linear mixed model adjusted for age and body mass index. The stability of density change at 1 year was assessed by evaluating mean squared error loss from predictions based on individual or mean density change at 1 year.

**Results:**

Analysis used mammograms from 126 healthy premenopausal women before and as they received tamoxifen for prevention (median age 42 years) and 172 matched controls (median age 41 years), with median 3 years follow-up. There was a strong correlation between percentage density measures used on the same mammogram in both the tamoxifen and no tamoxifen groups (all correlation coeficients > 0.8). Tamoxifen reduced mean breast density in year 1 by approximately 17–25% of the inter-quartile range of four automated percentage density measures at baseline, and from year 2, it decreased further by approximately 2–7% per year. Predicting change at 2 years using individual change at 1 year was approximately 60–300% worse than using mean change at 1year.

**Conclusions:**

All measures showed a consistent and large average tamoxifen-induced change in density over the first year, and a continued decline thereafter. However, these measures of density change at 1 year were not stable on an individual basis.

## Introduction

Tamoxifen is used in adjuvant settings to reduce the chance that breast cancer will reoccur when it has been diagnosed at an early stage, and also to slow disease progression in the advanced stage [1, 2]. It has been licensed for breast cancer prevention in healthy women at increased risk of the disease in several countries [3]. However, it might not be effective for prevention or treatment of all women and it has certain harms, including a slight increased risk of endometrial cancer, cataracts, pulmonary embolism and deep vein thrombosis. It would be useful to be able to better stratify women by risk in order to determine who will benefit most from the drug.

In the treatment setting, several prognostic factors for women diagnosed with breast cancer have been identified. These include classical factors such as tumour size, grade and lymph node involvement, and biomarkers including Ki67 and commercial genetic signatures such as OncotypeDX, ProSigna and EpiClin [4]. In the prevention setting, models are available to assess risk of breast cancer and may be used to help target preventive therapy with tamoxifen [5]. In both populations, there is evidence that mammographic density reduction associated with tamoxifen is a further prognostic factor [6–14]. This biomarker might help to personalise treatment and preventive therapy, suggesting different management when no reduction is seen. However, change in mammographic density associated with endocrine therapy is not currently routinely assessed nor incorporated into clinical decision-making.

An important gap in knowledge is how to measure breast density change associated with tamoxifen. Reliability is needed for baseline measurements to assess disease risk or the need for use of additional screening modalities, such as ultrasound, contrast-enhanced mammography or magnetic resonance imaging (MRI). Reliability is sometimes measured by comparing measurement error with the total variation in the sample (the ratio is equal to 1 minus the intra-class correlation coefficient). To help determine the clinical utility of breast density change associated with tamoxifen, we believe it is also important to assess the *stability* of measures of individual breast density change. Our definition of stability has not been used previously to test measures of breast density change, and so we introduce it next. It was developed following a so-called predictive sequential (or prequential) approach to inference [15]. This approach is more often applied to compare competing forecasting models [16]. Here, we test for lack of stability by determining whether using observed density change for a woman at 1 year is a better predictor of 2 years change than using the mean change for all women at 1 year (i.e. same prediction for all). We call any measure of density change that fails this predictive test unstable. We find it to be a worthwhile test because it would be hard to justify using an unstable measure of breast density as a basis to recommend individual women to stop therapy at 1 year.

The only study in the prevention setting to have shown a relationship between density change and incidence of breast cancer in healthy women receiving tamoxifen used visual assessment on a 5% point scale by a single reader [7]. Other research studies on density change in the treatment setting have also relied on visual assessment of breast density. In the adjuvant setting, this has been based on pairs or sequences of mammograms approximately 1 year apart [17–19]. In the tamoxifen-prevention setting, we are not aware of reports with more than two measurements. As most research has used visually assessed breast density change between two time points, it might be considered the current gold standard method. However, visual assessment is impractical for routine use and not reliable due to inter- and intra-reader variation and so more recent work has considered fully automated measures of breast density [20].

The main aim of this study was to determine the reliability, stability and size of different measures of mammographic density change in premenopausal women receiving tamoxifen for preventive therapy, and in comparison with a group who did not receive tamoxifen. To achieve this, we compared data from two cohorts of high-risk women in Manchester, UK: (i) those enrolled into a tamoxifen-prevention study (TAM-PREV) that was designed to determine uptake of tamoxifen in the target population and to investigate the possibility that we can target treatment to women most likely to benefit, and (ii) other women of the same age attending the same family-history clinic, but without receiving tamoxifen over the same period. Our objective was to assess average change in different measures of breast density over 5 years, and to determine the reliability and stability of individual mammographic density change in order to potentially guide clinical decisions.

## Methods

### Patients

Premenopausal women with no previous diagnosis of breast cancer and at high risk of breast cancer eligible for tamoxifen for prevention were invited to join the TAM-PREV study, which was designed to study uptake and acceptance of tamoxifen. Precise eligibility criteria are given in the supplementary material. Participants in TAM-PREV were given 20 mg/day of tamoxifen, orally for a maximum 5 years. They attended yearly follow-up appointments for mammography and where adherence was monitored via self-report in person. Of the 135 women who joined TAM-PREV, *n*=2 (1%) did not receive tamoxifen; *n*=30 (22%) started but stopped tamoxifen within 1 year, a further *n*=11 (8%), 17 (13%), 7 (5%) and 3 (2%), respectively, in 1-year intervals from 1.5 to 4.5 years; and *n*=76 (56%) completed the full 5 years. A cohort consisting of women attending the same family-history clinic who did not receive tamoxifen were also enrolled and consented in the FH-Risk study. All participants had a baseline mammogram over the same epoch (2010–2013) [21].

### Mammographic density

Full field digital mammography was used to screen women for breast cancer at each visit. Both raw (DICOM type ‘FOR PROCESSING’) and processed (‘FOR PRESENTATION’) mammograms of two views (medio-lateral oblique, MLO; cranial caudal, CC) of both breasts were obtained and used to measure breast density by different methods. The majority of mammograms were taken using GE machines (80%); others were on Hologic, Ims Giotto and Siemens Inspiration machines; in this analysis, only GE machines were used since one method (NN-VAS) is only designed for mammograms from this manufacturer. Four fully objective measures of breast density were computed on each of the images. They were (i) NN-VAS (neural network fitted to percentage density on visual assessment scale) version 1.0 [22], (ii) Volpara volumetric density version 1.5.2 [23], (iii) Stratus [24] and (iv) Densitas version 2.0.0 [20]. The mean of each density measure over all views and both breasts was the single measure used at each time point. Reliability of the different density measures was broadly similar, with intra-class correlation coefficients from 0.88 (Densitas) to 0.93 (Stratus) (Supplementary Table S8). To enable comparison with the results from the only study to show a relation to clinical outcome [7] in the prevention setting, we included the same visual percentage density change measure from that study as a ‘gold standard’ reference. Percentage density at baseline and first follow-up mammogram was determined visually by a single reader (RW). This was done on a semi-continuous scale between 0 and 100% in 5% increments, which is the same measure and expert reader (RW) as an earlier study from the IBIS-I trial [7]. To assess reproducibility, visual assessment was done on two separate occasions: (i) when using both cohorts and blinded to treatment allocation and order of mammograms, and (ii) on a subset of the tamoxifen cohort and unblinded to order of mammograms. This showed that visual assessment of a 10% or more percentage change was discordant for one quarter of those women assessed twice (26/100).

### Study design

Up to two women in the no tamoxifen (control) group were matched to the tamoxifen cohort (TAM-PREV), based on availability of matched controls defined by age at baseline mammogram (± 1 year) and year of baseline mammogram. All women had a mammogram at entry to the study (before tamoxifen use in the tamoxifen group) and were seen annually in the family-history clinic for up to 5 years for their routine follow-up appointment by AH (median 3 years in both groups). For women in the tamoxifen group, only mammograms during active treatment were used.

### Statistical analysis

Change in percentage density was the primary measure and in comparison with visually assessed percentage density change. Change in dense area and volume were assessed as secondary measures. The analysis only included time points where all automated density measures were available. This was done to ensure valid comparisons between the measures. A similar proportion of images were excluded for each method due to their internal quality control algorithms, except NN-VAS because only GE mammograms could be run (total mammograms *n*=1798; exclusions: Volpara *n*=72 (4%); Stratus *n*=124 (7%); Densitas *n*=101 (6%); NN-VAS *n*=387 (22%); total exclusions *n*=450 (25%)). Analysis used samples of women with mammographic density available (A) at baseline, (B) baseline and first year follow-up and (C) the subset of (B) additionally with breast density at year 2 follow-up.

Summary statistics were reported on the original breast density scale. All other analysis used transformed measures. Transformations were used to reduce skewness and improve approximation to normality. Volumetric percentage density and dense volume were natural logarithm transformed; percentage density measures square root transformed. To help make comparisons between the density methods, the transformed densities were standardised to have zero mean and unit inter-quartile range at baseline. Correlation between the density methods was assessed between women through a weighted Pearson correlation coefficient [25]. Cutpoints for density change for automated methods were selected to aid interpretation with respect to results from the IBIS-I trial [7]: density change in automated methods was defined by a cutpoint that yielded the same number of women in the no tamoxifen group above the cutpoint as the visual 10% absolute density change method and cutpoint used in the earlier study [7].

A normal linear mixed model for each density measure was fitted by maximum likelihood [26]. Fixed effects were included for (1) intercept, (2) group (tamoxifen vs no tamoxifen), (3) age (per 5 years), (4) body mass index (per 5 kg/m^2^), (5) time greater than 1 year (indicator variable), (6) year for year 2 onwards, (7) interaction between terms (2) and (5) and (8) interaction between (2) and (6). Random effects at the participant level were included to allow for different trajectories for each woman for a random intercept and slope (time since baseline mammogram), which were assumed bivariate normal. The fixed effects (5) and (7) allow for a different change in density at 1 year between the groups; (6) and (8) allow for different rates of change by year thereafter. Missing body mass index was imputed using the mean. Reliability was estimated using intra-class correlation coefficients (ICCs) from the model, being defined as the estimated residual variance divided by total variance. Profile likelihood 95% confidence intervals were obtained for the model fit parameters. A non-parametric empirical bootrap 95% confidence interval (3000 resamples) was used for the ICC statistics. In a sensitivity analysis, the model was re-fitted in the tamoxifen group alone (without fixed effects (2), (7) and (8)).

A predictive test was used to assess stability of different measures of breast density change at 1 year in women receiving tamoxifen. This compared predicting change at 2 years to be either (i) individual change at 1 year or (ii) mean density change at 1 year (i.e. the same predicted change in density for all women). Predictive ability was measured using mean squared error (MSE). This loss function is also the criterion used to fit ordinary linear regression models. The MSE of (i) relative to reference (ii) was estimated with non-parametric percentile bootstrap 95% confidence intervals.

All statistical analysis used software R 3.4.1 [27, 28]

## Results

**Fig. 1:**
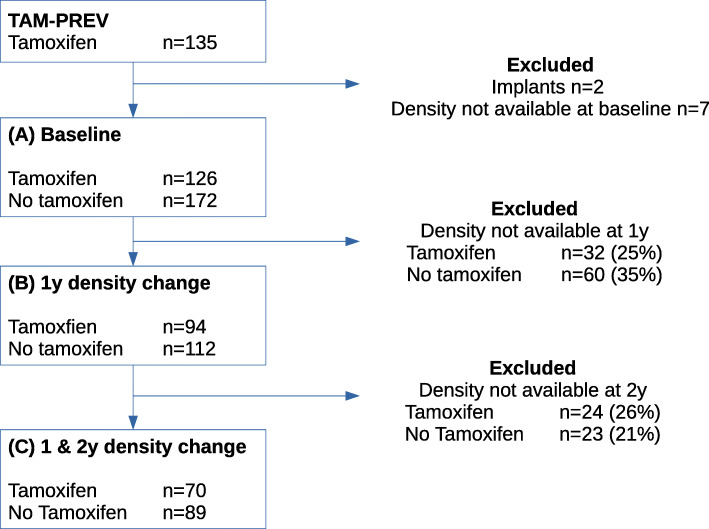
Flow diagram with number of women showing the number of women included in analysis samples A (all women included in the study), B (those with 1 year density change) and C (those with 1 year and 2 years density change measured)

The two cohorts were broadly comparable at baseline in analysis samples A (tamoxifen *n*=126, no tamoxifen *n*=172), B (tamoxifen *n*=94, no tamoxifen *n*=112) and C (tamoxifen *n*=70, no tamoxifen *n*=89; Fig. 1, Supplementary Tables S1-S5). The tamoxifen and no tamoxien groups were well matched for age and had similar body mass index. Approximately one quarter in both the tamoxifen and no tamoxifen group were obese (body mass index of greater than 30 kg/m^2^), one quarter over weight (body mass index greater than 25 kg/m^2^), and one quarter had a BMI less than approximately 22 kg/m^2^. Projected lifetime risk was similar, with the majority at high risk (more than 30% lifetime risk). Approximately 80% had children, and the age at first child was similar in those with children, being 27 years on average in the tamoxifen and no tamoxifen groups.

Mean percentage density differed substantially between all methods of measurement, as did the average change in percentage density between baseline and year 1 in the tamoxifen group. The mean absolute percent density change in women receiving tamoxifen ranged from approximately 1.4 (Volpara) to 7.8% (Stratus), while there was very little change during the first year in the no tamoxifen group (Table 1). However, there was a strong correlation between different percentage density measures in both the tamoxifen and no tamoxifen groups (all repeated-measures correlation coefficient > 0.8, Table 2 and Supplementary Table S6, Supplementary Figure S1), and on a standardised scale, the average change was similar for different measures of breast density. Total volume or area was negatively correlated with percentage density partly because it is the denominator of percentage density.

**Table 1:** Summary statistics of density measures at baseline (T0) and year 1 (T1) in analysis sample A (all women included in the study)

	Tamoxifen				No tamoxifen		
	*N* (unk%)	Median (IQR, range)	Mean (SD*)		*N* (unk%)	Median (IQR, range)	Mean (SD)
Visual % blinded							
Baseline	125 (1%)	25.0 (15.0–45.0, 0.0–90.0)	32.0 (21.7)		171 (1%)	30.0 (15.0–47.5, 0.0–80.0)	31.6 (20.6)
T1	98 (1%)	20.0 (10.0–40.0, 0.0–90.0)	26.0 (18.8)		122 (2%)	30.0 (15.0–50.0, 0.0–90.0)	33.5 (22.7)
T1–T0	94 (1%)	−5.0 (−13.8–0.0, −45.0–20.0)	−6.4 (9.8, −0.66)		112 (2%)	0.0 (−5.0–5.0, −25.0–30.0)	−0.2 (9.6)
Visual % unblinded							
Baseline	98 (22%)	25.0 (15.0–45.0, 0.0–95.0)	31.3 (20.5)		0 (100%)		
T1	96 (3%)	20.0 (10.0–30.0, 0.0–90.0)	23.4 (18.0)		0 (100%)		
T1–T0	92 (3%)	−5.0 (−12.8–5.0, −35.0–25.0)	−8.7 (9.4, −0.92)		0 (100%)		
Stratus %							
Baseline	126 (0%)	37.8 (13.5–56.8, 0.2–77.2)	34.6 (23.6)		172 (0%)	42.4 (9.1–58.0, 0.1–75.3)	36.2 (24.3)
T1	99 (0%)	27.6 (4.7–45.5, 0.1–67.4)	27.2 (21.3)		124 (0%)	42.7 (12.7–57.2, 0.1–71.0)	36.1 (23.5)
T1–T0	95 (0%)	−6.2 (−12.9–0.9, −56.5–12.0)	−7.8 (9.7, −0.81)		114 (0%)	−0.1 (−3.5–1.5, −27.7–56.5)	−0.2 (8.3)
Stratus DA							
Baseline	126 (0%)	47.8 (17.2–64.7, 0.6–167.6)	46.5 (34.8)		172 (0%)	44.4 (16.9–67.3, 0.3–211.2)	48.3 (39.3)
T1	99 (0%)	29.9 (9.1–47.7, 0.2–128.1)	34.2 (28.9)		124 (0%)	42.2 (20.2–69.9, 0.3–181.5)	48.7 (38.5)
T1–T0	95 (0%)	−10.8 (−19.2–3.3, −75.5–20.3)	−13.0 (13.7, −0.95)		114 (0%)	−1.0 (−6.3–3.2, −54.4–108.3)	−0.5 (14.3)
Densitas %							
Baseline	126 (0%)	41.0 (32.0–49.5, 12.0–73.0)	41.2 (12.9)		172 (0%)	42.0 (32.0–49.0, 10.0–72.0)	41.1 (12.3)
T1	99 (0%)	38.0 (29.0–47.0, 13.0–66.0)	37.7 (12.3)		124 (0%)	40.0 (34.0–50.2, 13.0–70.0)	41.4 (12.0)
T1–T0	95 (0%)	−5.0 (−8.0–1.5, −21.0–34.0)	−4.3 (7.0, −0.62)		114 (0%)	0.0 (−3.0–3.0, −21.0–25.0)	−0.4 (5.9)
Densitas DA							
Baseline	126 (0%)	44.0 (32.2–57.0, 15.0–131.0)	47.5 (22.3)		172 (0%)	41.5 (31.0–57.0, 19.0–162.0)	47.3 (24.0)
T1	99 (0%)	36.0 (27.5–49.5, 15.0–143.0)	41.4 (20.9)		124 (0%)	43.0 (33.0–55.2, 19.0–157.0)	48.5 (23.3)
T1–T0	95 (0%)	−6.0 (−11.0–3.0, −35.0–103.0)	−5.8 (14.0, -0.42)		114 (0%)	0.0 (−3.0–4.0, −42.0–33.0)	0.1 (8.2)
Volpara %							
Baseline	126 (0%)	8.6 (5.4–15.7, 2.2–31.4)	11.0 (6.8)		172 (0%)	9.5 (5.7–14.1, 2.2–29.0)	10.7 (6.2)
T1	99 (0%)	7.5 (4.7–13.0, 2.1–27.8)	9.3 (5.8)		124 (0%)	9.1 (5.7–15.4, 2.1–28.2)	11.0 (6.2)
T1–T0	95 (0%)	−0.9 (−2.3–0.2, −9.8–4.6)	−1.4 (2.5, −0.56)		114 (0%)	0.0 (−0.9–0.9, −13.9–10.4)	−0.2 (2.8)
Volpara DV							
Baseline	126 (0%)	61.4 (43.9–86.2, 17.6–189.9)	69.4 (36.2)		172 (0%)	62.1 (40.6–89.1, 14.4–246.8)	70.7 (38.8)
T1	99 (0%)	49.7 (35.9–67.0, 17.5–173.1)	55.3 (27.3)		124 (0%)	57.5 (41.1–85.2, 11.3–196.5)	67.7 (38.4)
T1–T0	95 (0%)	−11.0 (−21.6–2.7, −74.2–26.5)	−14.2 (16.3, −0.87)		114 (0%)	−2.5 (−11.3–4.5, −83.5–51.0)	−3.8 (18.7)
NN-VAS %							
Baseline	126 (0%)	41.1 (29.7–54.5, 7.1–76.8)	42.0 (16.3)		172 (0%)	43.8 (31.1–53.5, 8.4–76.0)	42.5 (16.1)
T1	99 (0%)	37.4 (23.6–47.0, 5.3–73.7)	36.9 (15.0)		124 (0%)	44.4 (31.2–54.2, 6.8–78.0)	42.5 (16.6)
T1–T0	95 (0%)	−5.3 (−8.8–2.0, −24.2–18.1)	−5.3 (5.8, −0.90)		114 (0%)	−0.7 (−3.6–1.0, −19.8–31.8)	−1.1 (6.0)

*DA* dense area, *DV* dense volume, *IQR* inter-quartile range, *SD* standard deviation, *unk* unknown

*T1–T0 for tamoxifen group statistic is mean (SD, mean/SD)

**Table 2:** Repeated measures correlation between density measures in the group of women that did not receive tamoxifen from analysis sample A (all women included in the study)

	Visual %	Stratus %	Densitas %	Volpara %	Stratus DA	Densitas DA	Volpara DV	Densitas BA	Volpara TV
Stratus %	0.85								
Densitas %	0.87	0.90							
Volpara %	0.89	0.92	0.92						
Stratus DA	0.68	0.86	0.74	0.67					
Densitas DA	0.12	0.14	0.18	−0.02	0.57				
Volpara DV	0.40	0.41	0.44	0.28	0.73	0.81			
Densitas BA	−0.45	−0.48	−0.51	−0.62	−0.03	0.74	0.42		
Volpara TV	−0.49	−0.52	−0.49	−0.69	−0.05	0.64	0.50	0.88	
NNVAS %	0.89	0.93	0.92	0.91	0.83	0.25	0.52	−0.39	−0.43

*DA* dense area, *DV* dense volume, *BA* breast area, *TV* total volume, *NNVAS* neural network visual assessment score

**Fig. 2:**
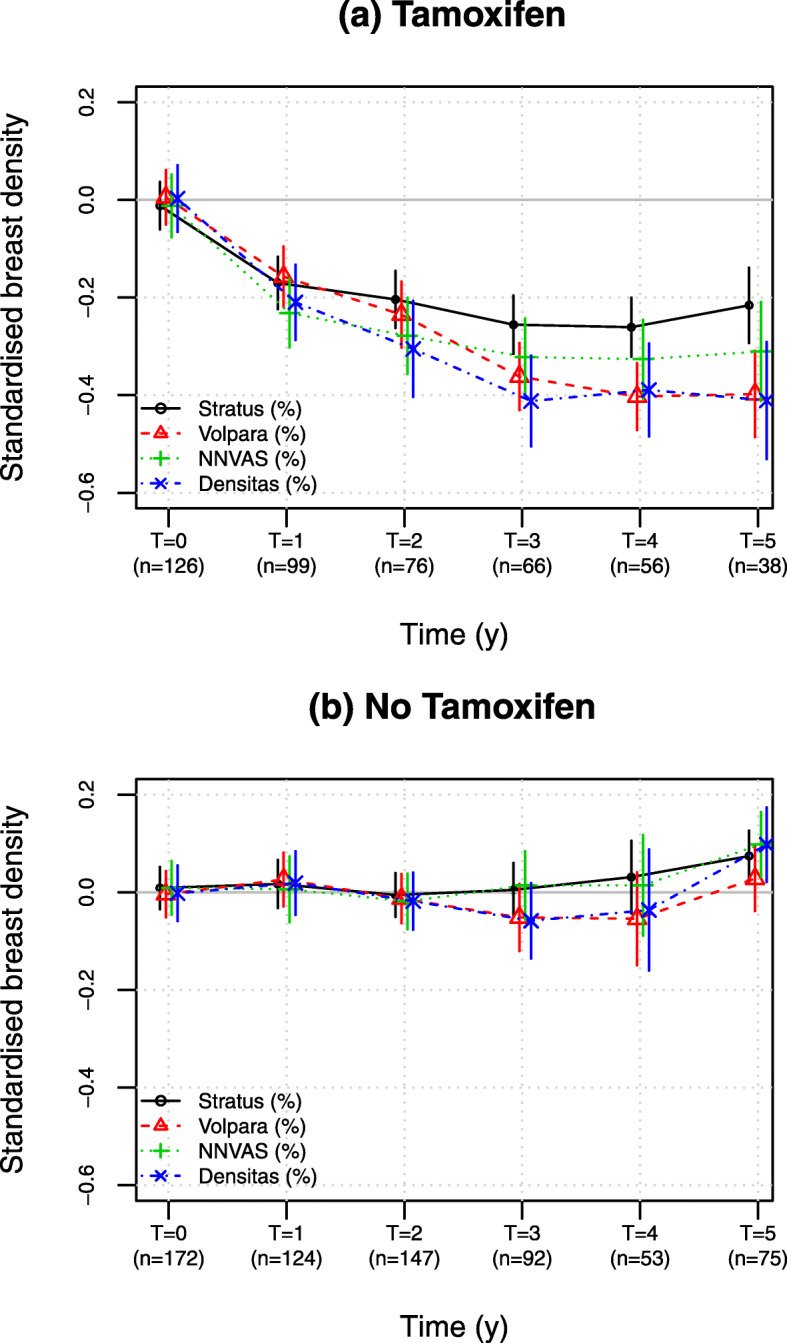
Mean (standard error) standardised breast density at each time point from women **a** receiving tamoxifen and **b** not receiving tamoxifen, using all women included in the study (analysis sample A). Standardised breast density uses transformed percentage density (natural logarithm for Volpara, square root for others) that has been normalised to have mean zero and unit inter-quartile range at baseline

Figure 2 shows an initial decline at 1 year followed by a sustained decrease thereafter in the tamoxifen group. This was also seen from age and BMI-adjusted estimates of density change. The estimated adjusted change at 1 year was approximately 17–25% of the IQR of each percentage density measure at baseline (Supplementary Table S7). The adjusted yearly decrease in density from year 2 was estimated to range from approximately 2 to 7% of IQR change per year depending on the density measure. The model fit was similar for each measure of breast density (Supplementary Tables S7 and S8). In a model fitted to the tamoxifen group alone, all methods except percentage density by Densitas had a moderate correlation between the random intercept and slope (approximately −0.3 to −0.6, Supplementary Tables S9, S10), indicating a larger tamoxifen-induced decrease in breast density for those with higher breast density at baseline.

Results from automated methods of density change were compared to visual assessment as the gold standard method. For a similar proportion of women who did not receive tamoxifen (approximately 16%), the automated methods identified a greater proportion of women in the tamoxifen group with change above their high cutpoint than visual assessment based on a 10% threshold (approximately 60% for automated compared with 37% for visual; Table 3). Of the 26 women with more than 10% density change based on visual assessment in the tamoxifen group, *n*=18 (Stratus and Volpara) to *n*=20 (Densitas and NN-VAS) were identified by automated methods. The automated measures all agreed on change at year 1 for *n*=19 (27%) of 70 women, but only 6 (9%) of 70 women at first follow-up did not fall into a high change group by any of the measures (Supplementary Table S11).

**Table 3:** Number of women with a decrease in breast density greater than a cutpoint using analysis sample C (women with density change measured at 1 year and 2 years)

	Cutpoint*	Cutpoint*	Tamoxifen				No tamoxifen			Decrease
Measure	no change	change	No change	Intermediate	Decrease		No change	Intermediate	Decrease	difference (95% CI)
(a) Visual %										
T1–T0	0.0	−10.0	24 (34%)	20 (29%)	26 (37%)		57 (64%)	18 (20%)	14 (16%)	21% (7–36%)
(b) Stratus %										
T1–T0	0.0	−4.6	10 (14%)	16 (23%)	44 (63%)		45 (51%)	30 (34%)	14 (16%)	47% (32–62%)
T2–T0	0.0	−4.6	9 (13%)	14 (20%)	47 (67%)		50 (56%)	20 (22%)	19 (21%)	46% (31–61%)
(c) Densitas %										
T1–T0	0.0	−3.6	9 (13%)	17 (24%)	44 (63%)		48 (54%)	27 (30%)	14 (16%)	47% (32–62%)
T2–T0	0.0	−3.6	7 (10%)	23 (33%)	40 (57%)		47 (53%)	24 (27%)	18 (20%)	37% (21–52%)
(d) Volpara %										
T1–T0	0.0	−1.4	14 (20%)	17 (24%)	39 (56%)		45 (51%)	30 (34%)	14 (16%)	40% (25–55%)
T2–T0	0.0	−1.4	5 (7%)	19 (27%)	46 (66%)		48 (54%)	22 (25%)	19 (21%)	44% (29–60%)
(e) NNVAS %										
T1–T0	0.0	−4.4	8 (11%)	24 (34%)	38 (54%)		36 (40%)	39 (44%)	14 (16%)	39% (23–54%)
T2–T0	0.0	−4.4	10 (14%)	14 (20%)	46 (66%)		47 (53%)	29 (33%)	13 (15%)	51% (37–66%)

T0, density at entry; T1, density at 1 year; T2, density at 2 year; no change, density change greater than or equal to this is classed as no change; change, density change less than or equal to this is classed as decrease; the intermediate category is density in between these; decrease difference, absolute difference in density change percentage between tamoxifen and no tamoxifen groups

*For automated methods, the cutpoints shown are for differences in transformed percentage density (×10), being the natural logarithm for Volpara and square root for the other methods.

While similar average changes were detected, individual breast density change at 1 year was not stable for 2 years change using any measure. Predicting each woman’s density change at 2 years to be that observed at 1 year had between approximately 60 to 300% greater error than using the overall mean density change at 1 year (Table 4). The lack of stability in measurement of individual breast density change was further indicated by disagreement in women above a threshold using the same density measure of change based on change at 1 year or 2 years for approximately 40% women (Supplementary Table S12).

**Table 4:** Mean squared error (×100) in analysis sample C (women with density measured at 1 year and 2 years) {relative performance (95% CI)} for predicting density change at 2 years, using mean density change at year 1 (REF = reference predictor) or individual observed density change at year 1

	Stratus %	Densitas %	Volpara %	NNVAS %
Mean change T0–T1	6.0 {REF}	21.4 {REF}	4.2 {REF}	12.0 {REF}
Individual change T0–T1	14.5 {2.4 (1.6 to 3.3)}	65.9 {3.1 (1.8 to 3.9)}	10.0 {2.4 (1.8 to 3.0)}	19.7 {1.6 (1.3 to 2.3)}

*T0* entry, *T1* follow-up at 1 year

## Discussion

This study investigated five measures of mammographic density in women receiving tamoxifen preventive therapy, compared with similar women who did not, over up to 5 years. The primary focus was on change at 1 year. There were two main findings.

Firstly, all breast density measures revealed a large average change in density among women receiving tamoxifen for prevention over the first year, and a continued average decline thereafter. The size of these changes in comparison with a no tamoxifen group was consistent between the different measures when assessed on standardised scales. This appears to be the first report in a preventive setting to demonstrate there is a continued decline in mammographic density beyond the first year of receiving tamoxifen.

Secondly, even if breast density change over 1 year is a prognostic or predictive biomarker for women receiving tamoxifen for breast cancer prevention, current measures of mammographic density might not measure change in breast density at 1 year with sufficient stability for clinical use. The analysis indicates that if a clinician advises a woman that her breast density has changed by the amount measured at 1 year, then on aggregate this may be up to 300% worse (using mean squared error) as a prediction of her 2 years breast density change than advising all women that their density is likely to have changed by the same amount. It therefore appears that mammographic density change at 1 year is not stable using current technology.

The standardised and transformed scales used alter interpretation of the density change on the original scale. The change in logarithm volumetric density is equivalent to a relative change (ratio). The difference between square root percentage density is less interpretable, but is partly justified because on these transformed and standardised scales all measures showed similar average effects of tamoxifen through time, even though they are all different algorithms. There were some apparent subtle differences, such as Stratus showing a smaller decrease in standardised density than the others (Fig. 2, Supplementary Table S7), but we are cautious about over interpretation of such differences. For example, we would observe a different point estimate if we had applied a different transformation than square root for Stratus (c.f. Supplementary Figure S1).

Overall results across the different density methods were similar and in agreement with a gold standard method based on visual assessment. The similar average effects and reliability (ICCs) suggest merit for all in clinical studies designed to measure average change in breast density. The similarity between methods is striking because there are substantial differences between how the algorithms function. The volumetric method (Volpara) is based on a medical physics model. The other methods use machine learning algorithms. Stratus includes an algorithm to align images so that density change is more stable. NN-VAS was trained on visual assessment of raw unprocessed images from the PROCAS study [22], which was from the same Manchester setting as the present study. Densitas uses processed DICOM files.

Despite agreement between measures on average, we found that density change at an individual level was not stable. This might be caused by other factors than the breast density algorithms. Alternative explanations include possible natural variation in breast density, such as during the menstrual cycle. Another possible explanation is variability in the way mammography might be done through time. Further research could try to identify how to make density change measures more stable. The net result of this is that in settings that are similar to the current study, it seems inappropriate to recommend women to end tamoxifen after a year of use, solely on the basis that their breast density has not decreased. Lack of change at 1 year might be due to measurement error. On the flip side, if a woman has observed a large breast density change at 1 year, then adherence could be encouraged if findings from the single research study on breast density change and breast cancer incidence are replicated in the future [7]. This is because they suggest that, on average, a group of women with larger density changes appear to have better prognosis. Similar reasoning would apply in a tamoxifen treatment context.

This study has a number of limitations. Firstly, due to the study size, we were unable to link change in density due to tamoxifen with subsequent risk reductions in breast cancer. Secondly, treatment was not randomised between the tamoxifen and no tamoxifen groups. Thirdly, we were unable to assess what happened to breast density once tamoxifen stopped being prescribed. Fourthly, weight and menopausal status were not monitored through time for all women. Knowing about these changes through time would enable a more precise determination of breast density change since they are established to be associated with breast density.

Strengths of this study include that compliance was actively monitored yearly, and we excluded breast density after drug use ended. This ensured an accurate measure of the effect of tamoxifen on mammographic density, but is also important for prognosis studies of breast density change. For example, a potential bias in studies of breast density change and prognosis in women who have been recommended to receive tamoxifen is that it could partly reflect compliance: those who benefit are those who received the drug.

In summary, on the aggregate level (e.g. evaluating an entire population or clinical trial), tamoxifen-associated density declines may provide an important signal (e.g. for indicating tamoxifen effectiveness) but this signal appears to be ‘too noisy’ at the individual level to use it to make clinical recommendations. We expect these findings would be generalisable to treatment settings as well, but further studies are warranted to test this hypothesis.

## Conclusions

Our study provides the first analysis in a tamoxifen-prevention setting of longitudinal breast density measured yearly over up to 5 years, and includes fully automated measures that have not previously been examined in a tamoxifen prevention or treatment context. The data provide important information on the reliability, stability and size of breast density changes from different methods used to measure breast density. All measures showed a large average decline in breast density after 1 year of tamoxifen, and a continued decline in mammographic density beyond the first year of receiving tamoxifen. All methods had relatively high intra-class correlation coefficients (approximately 0.9) indicating a good degree of reliability. However, breast density change at 1 year did not appear to be sufficiently stable to inform personalisation of clinical decisions on the basis of a woman’s observed breast density change. These findings will be important to clinicians and their patients undergoing or considering endocrine therapy, as well as regulators and ethics boards considering trials of products that require information on the reliability and stability of mammographic density reductions as an endpoint.

## Supplementary information

**Additional file 1** Supplementary material

## Data Availability

The dataset used and analysed during the current study is available from the corresponding author on reasonable request.

## References

[1] Rugo HS, Rumble RB, Macrae E, Barton DL, Connolly HK, Dickler MN, Fallowfield L, Fowble B, Ingle JN, Jahanzeb M, Johnston SRD, Korde LA, Khatcheressian JL, Mehta RS, Muss HB, Burstein HJ (2016). Endocrine therapy for hormone receptor-positive metastatic breast cancer: American Society of Clinical Oncology Guideline. J Clin Oncol.

[2] Davies C, Godwin J, Gray R, Clarke M, Cutter D, Darby S, McGale P, Pan HC, Taylor C, Wang YC, Dowsett M, Ingle J, Peto R, Early Breast Cancer Trialists’ Collaborative Group (2011). Relevance of breast cancer hormone receptors and other factors to the efficacy of adjuvant tamoxifen: patient-level meta-analysis of randomised trials. Lancet.

[3] Cuzick J, Sestak I, Bonanni B, Costantino JP, Cummings S, DeCensi A, Dowsett M, Forbes JF, Ford L, LaCroix AZ, Mershon J, Mitlak BH, Powles T, Veronesi U, Vogel V, Wickerham DL, SERM Chemoprevention of Breast Cancer Overview Group (2013). Selective oestrogen receptor modulators in prevention of breast cancer: an updated meta-analysis of individual participant data. Lancet.

[4] Buus R, Yeo B, Brentnall AR, Klintman M, Cheang MCU, Khabra K, Sestak I, Gao Q, Cuzick J, Dowsett M (2018). Novel 18-gene signature for predicting relapse in ER-positive, HER2-negative breast cancer. Breast Cancer Res.

[5] van Veen EM, Brentnall AR, Byers H, Harkness EF, Astley SM, Sampson S, Howell A, Newman WG, Cuzick J, Evans DGR (2018). Use of single-nucleotide polymorphisms and mammographic density plus classic risk factors for breast cancer risk prediction. JAMA Oncology.

[6] Kanbayti IH, Rae WID, McEntee MF, Ekpo EU (2019). Are mammographic density phenotypes associated with breast cancer treatment response and clinical outcomes? A systematic review and meta-analysis. Breast.

[7] Cuzick J, Warwick J, Pinney E, Duffy SW, Cawthorn S, Howell A, Forbes JF, Warren RML (2011). Tamoxifen-induced reduction in mammographic density and breast cancer risk reduction: a nested case–control study. J Natl Cancer Inst.

[8] Kim J, Han W, Moon H-G, Ahn SK, Shin H-C, You J-M, Han S-W, Im S-A, Kim T-Y, Koo HR, Chang JM, Cho N, Moon WK, Noh D-Y (2012). Breast density change as a predictive surrogate for response to adjuvant endocrine therapy in hormone receptor positive breast cancer. Breast Cancer Res.

[9] Knight JA, Blackmore KM, Fan J, Malone KE, John EM, Lynch CF, Vachon CM, Bernstein L, Brooks JD, Reiner AS, Liang X, Woods M, Bernstein JL, WECARE Study Collaborative Group (2018). The association of mammographic density with risk of contralateral breast cancer and change in density with treatment in the WECARE study. Breast Cancer Res.

[10] Ko KLL, Shin ISS, You JYY, Jung S-YY, Ro J, Lee ESS (2013). Adjuvant tamoxifen-induced mammographic breast density reduction as a predictor for recurrence in estrogen receptor-positive premenopausal breast cancer patients. Breast Cancer Res Treat.

[11] Li J, Humphreys K, Eriksson L, Edgren G, Czene K, Hall P (2013). Mammographic densitreduction is a prognostic marker of response to adjuvant tamoxifen therapy in postmenopausal patients with breast cancer. J Clin Oncol.

[12] Nyante SJ, Sherman ME, Pfeiffer RM, de Gonzalez A, Brinton LA, Aiello Bowles EJ, Hoover RN, Glass A, Gierach GL (2015). Prognostic significance of mammographic density change after initiation of tamoxifen for ER-positive breast cancer. J Natl Cancer Inst.

[13] Sandberg MEC, Li J, Hall P, Hartman M, Dos-Santos-Silva I, Humphreys K, Czene K (2013). Change of mammographic density predicts the risk of contralateral breast cancer - a case-control study. Breast Cancer Res.

[14] van Nes JGH, Beex LVAM, Seynaeve C, Putter H, Sramek A, Lardenoije S, Duijm-de Carpentier M, Van Rongen I, Nortier JWR, Zonderland HM, van de Velde CJH (2015). Minimal impact of adjuvant exemestane or tamoxifen treatment on mammographic breast density in postmenopausal breast cancer patients: a Dutch TEAM trial analysis,. Acta Oncol.

[15] Dawid AP (1984). Present position and potential developments: some personal views statistical theory the prequential approach. J R Stat Soc Ser A Gen.

[16] Brentnall AR, Crowder MJ, Hand DJ (2010). Predictive-sequential forecasting system development for cash machine stocking. Int J Forecast.

[17] Nyante SJ, Sherman ME, Pfeiffer RM, de Gonzalez AB, Brinton LA, Bowles EJA, Hoover RN, Glass A, Gierach GL (2016). Longitudinal change in mammographic density among ER-positive breast cancer patients using tamoxifen. Cancer Epidemiol Biomark Prev.

[18] Engmann NJ, Scott CG, Jensen MR, Ma L, Brandt KR, Mahmoudzadeh AP, Malkov S, Whaley DH, Hruska CB, Wu FF, Winham SJ, Miglioretti DL, Norman AD, Heine JJ, Shepherd J, Pankratz VS, Vachon CM, Kerlikowske K (2017). Longitudinal changes in volumetric breast density with tamoxifen and aromatase inhibitors. Cancer Epidemiol Biomark Prev.

[19] Eriksson L, He W, Eriksson M, Humphreys K, Bergh J, Hall P, Czene K (2018). Adjuvant therapy and mammographic density changes in women with breast cancer. JNCI Cancer Spectr.

[20] Astley SM, Harkness EF, Sergeant JC, Warwick J, Stavrinos P, Warren R, Wilson M, Beetles U, Gadde S, Lim Y, Jain A, Bundred S, Barr N, Reece V, Brentnall AR, Cuzick J, Howell T, Evans DG (2018). A comparison of five methods of measuring mammographic density: a case-control study. Breast Cancer Res.

[21] Evans DG, Astley S, Stavrinos P, Harkness E, Donnelly LS, Dawe S, Jacob I, Harvie M, Cuzick J, Brentnall A, Wilson M, Harrison F, Payne K, Howell A (2016). Improvement in risk prediction, early detection and prevention of breast cancer in the NHS Breast Screening Programme and family history clinics: a dual cohort study. Program Grants Appl Res.

[22] Ionescu GV, Fergie M, Berks M, Harkness EF, Hulleman J, Brentnall AR, Cuzick J, Evans DG, Astley SM (2019). Prediction of reader estimates of mammographic density using convolutional neural networks. J Med Imaging.

[23] Highnam R, Brady S, Yaffe M, Karssemeijer N, Harvey J, Martì J, Oliver A, Freixenet J, Martì R (2010). Robust Breast Composition Measurement - VolparaTM. International workshop in digital mammography.

[24] Eriksson M, Li J, Leifland K, Czene K, Hall P (2018). A comprehensive tool for measuring mammographic density changes over time. Breast Cancer Res Treat.

[25] Bland JM, Altman DG (1995). Calculating correlation coefficients with repeated observations: part 2–correlation between subjects. BMJ.

[26] Crowder MJ, Hand DJ (1990). Analysis of Repeated Measures.

[27] R Core Team (2017). R: a language and environment for statistical computing.

[28] Bates D, Mächler M, Bolker B, Walker S (2015). Fitting linear mixed-effects models using lme4. J Stat Softw.

