# Supplementary material for: Mammographic density change in a cohort of premenopausal women receiving tamoxifen for breast cancer prevention over 5 years
Source: Breast Cancer Res. 2020 Sep 29;22:101. doi: 10.1186/s13058-020-01340-4 (PMC7523310; doi:10.1186/s13058-020-01340-4)
Supplement: Supplementary file 1 — Additional file 1 Supplementary material [file 13058_2020_1340_MOESM1_ESM.pdf]

Additional Files

Additional file 1 — Supplementary material

Supplementary material captions

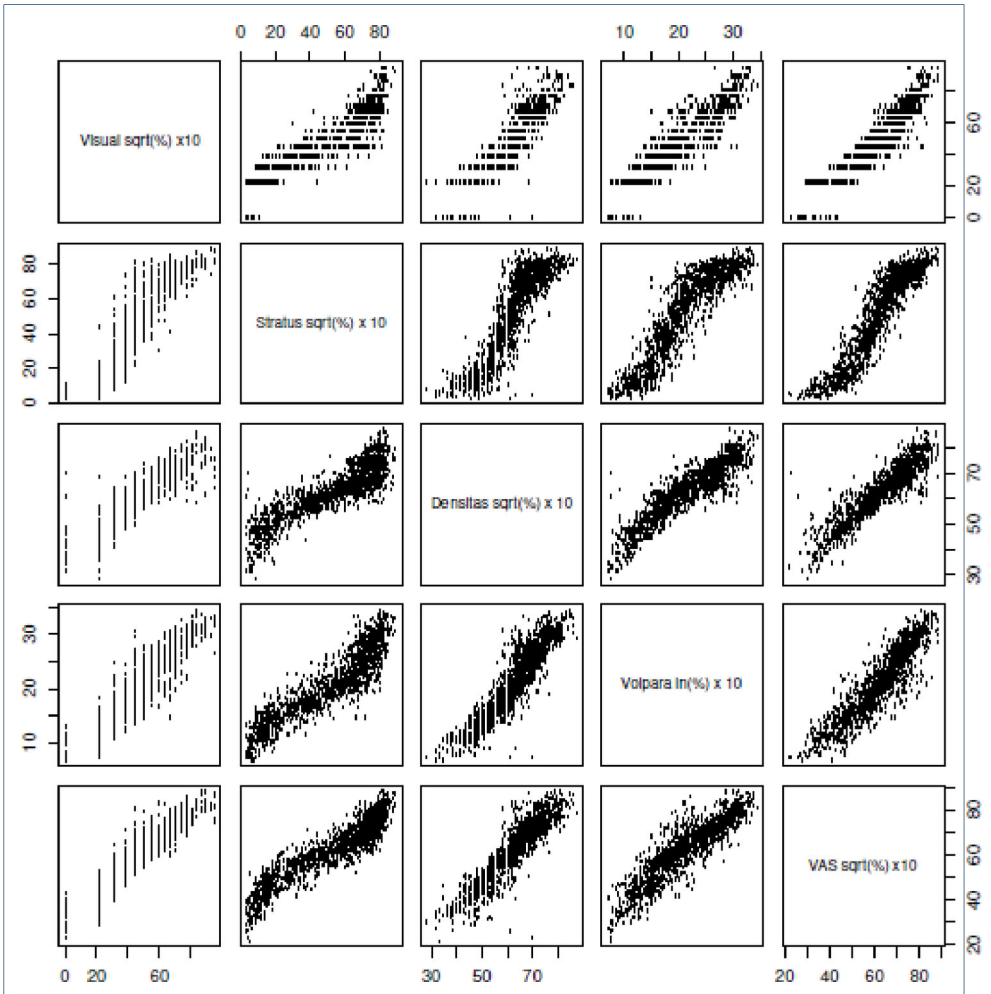

Figure S1 Matrix scatter plot to compare transformed percent density measures. Visual, Status, Densitas and NNVAS were square root transformed; the natural logarithm was used for Volpara.

Table S1 Baseline characteristics of participants in analysis sample A (all women included in the study).

Table S2 Baseline characteristics of participants in analysis sample B (women with 1y density change measured). Footnote: *summary gives median (interquartile range, range) for continuous variables and n (%) for parous; TP: Tamoxifen; FH: No Tamoxifen

Table S3 Baseline characteristics of participants excluded from analysis sample B (women with 1y density change measured). Footnote: *summary gives median (interquartile range, range) for continuous variables and n (%) for parous; TP: Tamoxifen; FH: No Tamoxifen

Table S4 Baseline characteristics of participants in analysis sample C (women with 1y and 2y density change measured). Footnote: *summary gives median (interquartile range, range) for continuous variables and n (%) for parous; TP: Tamoxifen; FH: No Tamoxifen

Table S5 Baseline characteristics of participants excluded from analysis sample C (women with 1y and 2y density change), out of all women included in the study (analysis sample A). Footnote: *summary gives median (interquartile range, range) for continuous variables and n (%) for parous; TP: Tamoxifen; FH: No Tamoxifen

Table S6 Repeated measures correlation (between women) between density measures for tamoxifen group, analysis sample A (all women included in the study).

Table S7 Linear mixed model fit, main effect estimates (95%CI)

Table S8 Linear mixed model fit estimated variance components (95%CI).

Table S9 Linear model fit, fixed effects (95%CI), tamoxiden group only.

Table S10 Linear mixed model fit estimated variance components (95%CI), tamoxifen group only.

Table S11 Number of women and total number of measures that agree on breast density measure change (Volpara %, Stratus %, Densitas %, NNVAS %) above cutpoint at year 1 (T0) or year 2 (T2) compared with baseline (T0).

Table S12 Agreement in density change between pairs of density measures (n=70 in total from tamoxifen group, analysis sample (C)). (a) compares between baseline (T0) and first followup at one year (T1); (b) compares change between baseline and two years later (T2); and (c) considers the concordance between change measured at one year (T0 - T1) and two years (T0 - T2), including for the same measure of breast density.
